# Learning to speciate: The biased learning of mate preferences promotes adaptive radiation

**DOI:** 10.1111/evo.12797

**Published:** 2015-10-26

**Authors:** R. Tucker Gilman, Genevieve M. Kozak

**Affiliations:** ^1^ Faculty of Life SciencesUniversity of Manchester; ^2^ Biology DepartmentTufts University

**Keywords:** Adaptive radiation, biased learning, mate preference learning, model, sexual imprinting, speciation

## Abstract

Bursts of rapid repeated speciation called adaptive radiations have generated much of Earth's biodiversity and fascinated biologists since Darwin, but we still do not know why some lineages radiate and others do not. Understanding what causes assortative mating to evolve rapidly and repeatedly in the same lineage is key to understanding adaptive radiation. Many species that have undergone adaptive radiations exhibit mate preference learning, where individuals acquire mate preferences by observing the phenotypes of other members of their populations. Mate preference learning can be biased if individuals also learn phenotypes to avoid in mates, and shift their preferences away from these avoided phenotypes. We used individual‐based computational simulations to study whether biased and unbiased mate preference learning promotes ecological speciation and adaptive radiation. We found that ecological speciation can be rapid and repeated when mate preferences are biased, but is inhibited when mate preferences are learned without bias. Our results suggest that biased mate preference learning may play an important role in generating animal biodiversity through adaptive radiation.

Adaptive radiations have produced spectacular examples of biodiversity, including the rift lake cichlids (Allender et al. 2003), Caribbean anoles (Losos 2009), and Galapagos finches (Grant and Grant 2014). However, despite their importance in evolutionary biology, we still do not know why adaptive radiations occur in some lineages and not in others (Schluter 2000; Gavrilets and Losos 2009; Losos 2009). During adaptive radiations, reproductive isolation evolves between populations as they diverge to fill different ecological niches in a process called ecological speciation (Schluter 2000; Nosil 2012). Because hybrids between recently diverged ecotypes are usually viable and fertile, the evolution of assortative mating is a critical step in this process (Schluter 2000; Allender et al. 2003; Coyne and Orr 2004; Nosil 2012). Understanding what causes assortative mating to evolve rapidly and repeatedly in the same lineage is key to understanding adaptive radiation (Nosil 2012).

Assortative mating in nature can be caused by different mechanisms. For example, it can occur when individuals with similar phenotypes use similar habitats (Dambroski et al. 2005; Snowberg and Bolnick 2012). Mate preferences can also lead to assortative mating, provided that individuals prefer mates with phenotypes similar to their own (Verzijden et al. 2005; Mavarez et al. 2006). Mate preferences can be genetically determined (Saether et al. 2007), but they can also be learned (Verzijden et al. 2012). For example, in sticklebacks females learn to prefer mates with phenotypes similar to their fathers (Kozak et al. 2011) and in some cichlids females learn to prefer mates with phenotypes similar to their mothers (Verzijden et al. 2008). Recent work suggests that learned preferences for parental phenotypes can promote speciation (Verzijden et al. 2005; Servedio and Dukas 2013).

Mate preferences in nature can be learned with bias (sometimes called “peak shift”; ten Cate et al. 2006; ten Cate and Rowe 2007). In this case, individuals have a target phenotype that they seek to match and an avoided phenotype that they seek to avoid in mates. The phenotype an individual prefers most strongly is shifted away from its target phenotype in the direction opposite its avoided phenotype (Fig. 1). For example, a female zebra finch seeks mates with her father's beak color and avoids mates with her mother's beak color. As a result, she tends to choose mates with beak colors more extreme than her father in the direction opposite her mother (ten Cate et al. 2006). Biased mate preferences can drive the evolution of extreme phenotypes, and in some cases cause runaway selection (Aoki et al. 2001; Kokko and Brooks 2003). Researchers have speculated that biased mate preference learning might facilitate speciation (Irwin and Price 1999; ten Cate and Rowe 2007; Verzijden et al. 2012), but this has not been studied.

**Figure 1:**
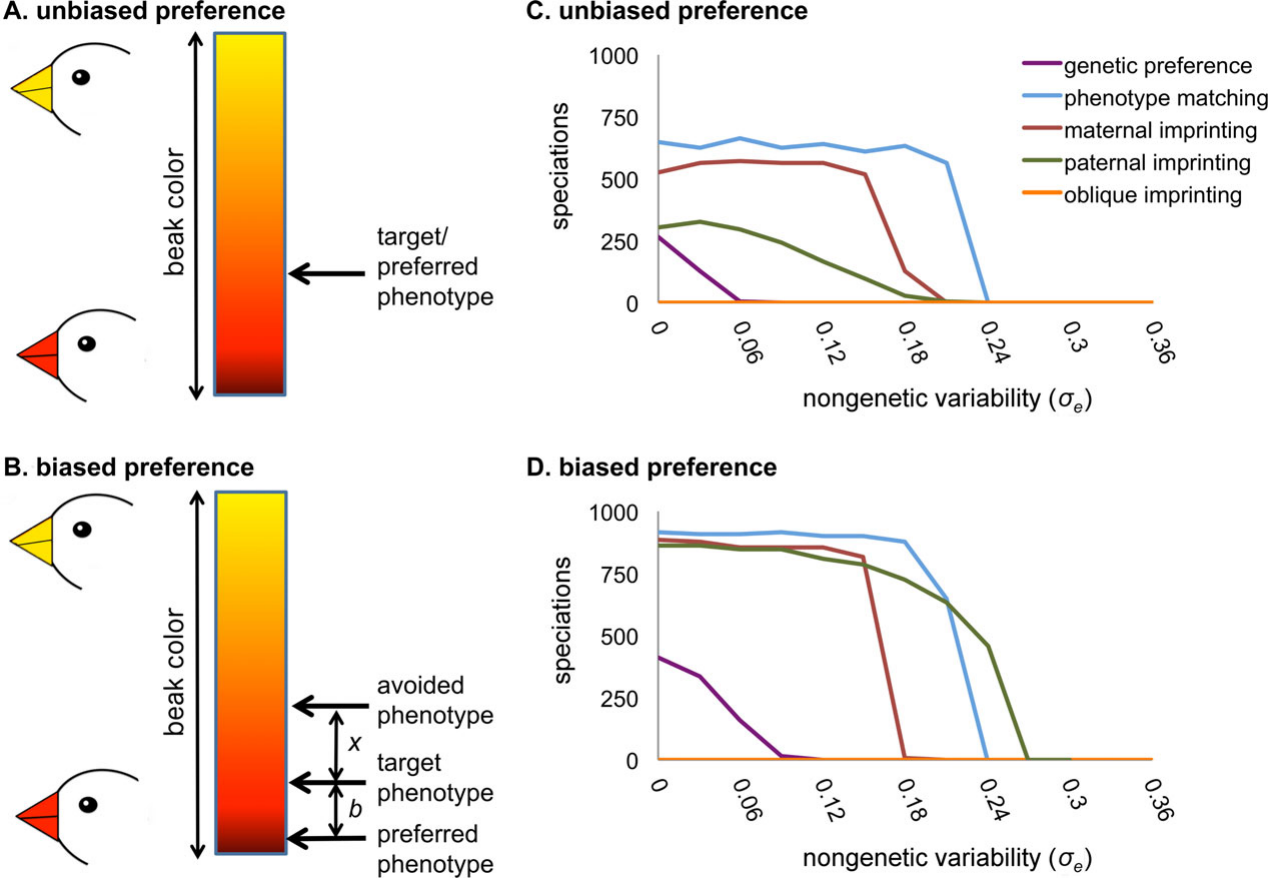
Biased mate preferences promote ecological speciation. Left panels illustrate unbiased (A) and biased (B) mate preferences. Color bars represent a continuous ecological trait, here beak color. When preferences are unbiased (A), females prefer mates with the target phenotype. When preferences are biased (B), the preferred phenotype is shifted away from the target phenotype by an amount *b* in the direction opposite the avoided phenotype. As in nature (ten Cate and Rowe 2007), *b* declines as the difference between the target and avoided phenotypes (*x*) increases. Right panels show speciations per 1000 simulations under each mate preference mode when preferences are unbiased (C) or biased away from an obliquely imprinted phenotype (D). Biased learning increases the probability of ecological speciation and expands the range of conditions under which speciation can occur.

We used stochastic individual‐based simulations to study how biases in mate preference learning influence the evolution of reproductive isolation (i.e., speciation) by assortative mating. Simulations began with randomly mating populations under disruptive ecological selection. Populations had the capacity to evolve some form of genetic or learned mate preference. If a mate preference evolved and assortative mating split the population into reproductively isolated groups (i.e., species), then we introduced an aliquot from one daughter species into a new environment where it was again under disruptive selection. For example, this might simulate the invasion of a new island by a population that evolved elsewhere (e.g., Caribbean anoles, Losos 2009). We asked whether the invading population speciated again, and if so how quickly respeciation occurred. This focus on repeated speciation makes our approach more suited to studying adaptive radiation than previous studies that have considered only single speciation events. We found that respeciation is rapid when mate preferences are biased away from a learned phenotype, but is rare and slow when mate preferences are unbiased. Thus, the biased learning of mate preferences may play a key role in rapid repeated speciation and in adaptive radiation in animals.

## Model

### TRAIT ARCHITECTURE

We modeled diploid populations of females and males. Each female expresses an ecological phenotype, a mate preference phenotype, and a mate choosiness phenotype. Males express the ecological phenotype, but because mating is by female choice they do not express mate preference or choosiness phenotypes.

The ecological phenotype, *z*, determines the resources that an individual can exploit. Examples of ecological phenotypes in nature include habitat preference (which determines the resources an individual encounters) and gape width (which determines the size of the food particles an individual can ingest; Hambright 1991; Malmquist et al. 1992). Each individual's ecological phenotype is represented by a single real number. The ecological phenotype includes genetic and nongenetic (i.e., environmental) components (*z_g_* and *z_e_*, respectively, where *z_g_* + *z_e_* = *z*). The genetic component is governed by 16 additive diploid loci, each of which houses one of an infinite number of possible real‐valued alleles. New alleles are created in each generation by mutation (as in Kimura and Crow 1964). Biologically, loci in our model can be thought of as quantitative trait loci (QTLs) and mutations can be thought of as single nucleotide replacements within QTLs (Kopp and Hermisson 2006). The nongenetic component of each individual's ecological phenotype (*z_e_*) is drawn independently from a distribution *N*(0,$\sigma_{e}^{2}$). Increasing σ*_e_* weakens the effect of selection on the ecological genotype and alters the strength of competition between individuals with different ecological genotypes.

A female's mate preference phenotype, *p*, describes the ecological phenotype she prefers in mates. When mate preferences are unbiased, each female prefers mates that match her target phenotype *p_t_* (Fig. 1A). We considered five different mechanisms (*modes*) by which females might acquire target phenotypes. When the mate preference mode is *genetic*, the target phenotype is controlled by 16 additive diploid loci, each of which houses one of an infinite number of real‐valued alleles. Genetically determined mate preferences are intrinsic (i.e., not learned). When the mate preference mode is *phenotype matching*, females prefer mates with ecological phenotypes similar to their own (i.e., a female's target phenotype equals her ecological phenotype). In nature, phenotype matching may be learned. For example, the ecological phenotype might determine the foraging habitat, and females might learn preferred phenotypes by observing other individuals at foraging sites. When the mate preference mode is *maternal imprinting* or *paternal imprinting*, each female learns a preference for her mother's or her father's phenotype, respectively. When the mate preference mode is *oblique imprinting*, each female learns a preference for the phenotype of an adult male that she randomly selects from her parents’ generation. Imprinted preferences always are learned.

Mate preference modes in our model can be biased. We call the combination of a mate preference mode with either bias or no bias a *mate choice strategy*. If the mate choice strategy is biased, then each female has both a target phenotype, *p_t_*, and an avoided phenotype, *p_a_*. Her preferred phenotype is shifted away from *p_t_* in the direction opposite *p_a_* (Fig. 1B). Females of many species assess potential mates relative to other males that they have observed (Gibson and Langen 1996; Rebar et al. 2011). Results presented below assume that females acquire avoided phenotypes by oblique imprinting (i.e., each female shifts her preference away from the phenotype of a randomly selected adult male from her parents’ generation). Results are qualitatively similar if females learn avoided phenotypes in other ways (e.g., by maternal or paternal imprinting; Supporting Information). In nature, bias is greatest when the target and avoided phenotypes are similar (ten Cate and Rowe 2007). We assumed that there is a maximum bias *b*_max_, and that the magnitude of bias declines linearly to zero as the absolute difference between *p_t_* and *p_a_* increases. If *p_t_* = *p_a_*, we assumed that the female ignores the avoided phenotype and there is no bias in mate preference. (Because the avoided phenotypes include a nongenetic components drawn from a continuous distribution, this rarely happens.) The bias in a female's mate preference is thus:

(1) bpt,pa= max b max 1−mb|pt−pa|,0 sgn pt−pa

where *m_b_* controls how quickly bias declines with |*p_t_* – *p_a_*|, and the function sgn returns the sign of its argument. The female's preferred phenotype is:

(2) $$p\left(p_{t},p_{a}\right)=p_{t}+b\left(p_{t},p_{a}\right).$$

A female's mate choosiness phenotype determines the strength of her mate preference. If a female with high choosiness encounters a potential mate with an ecological phenotype different from the one she prefers, she is likely to reject him. A female with low choosiness is less likely to do so, and a female with choosiness of zero or less than zero mates at random. Choosiness is governed by four additive diploid loci, each of which houses one of an infinite number of real‐valued alleles. The smaller number of choosiness loci than ecological loci reflects our assumption that fewer genes affect choosiness than affect complex ecological phenotypes in nature. Allowing negative choosiness alleles in our model ensures that mutation alone does not force populations to become choosy. This would bias simulations toward the evolution of assortative mating and therefore speciation.

### ENVIRONMENT

The environment in our model comprises the distribution of resources used by the population. Resources vary so that individuals with different ecological phenotypes are better able to use different resources. For example, if the ecological trait is gape width, then resources might vary in particle size. The function *K*(*z*) describes the resource distribution. Specifically, *K*(*z*) is the carrying capacity when all individuals have ecological phenotype *z*. *K*(z) takes the form:

(3) $$K\left(z\right)=K\left(z^{*}\right)\;\exp\left(-\frac{{\left|z-z^{*}\right|}^{\beta }}{2\sigma_{z}^{\beta }}\right),$$

where σ*_z_* controls the variability of the resources, β controls the shape (i.e., the kurtosis) of the resource distribution, and z* is the optimal ecological phenotype for a monomorphic population. Resource distributions with β ≥ 2 are biologically plausible and have been studied previously (Doebeli et al. 2007).

### DYNAMICS

We tracked populations through discrete generations that comprise viability selection and mating. Each generation begins with a population of juveniles that undergoes frequency‐dependent viability selection due to resource competition. Individuals compete most strongly with other individuals that have similar ecological phenotypes. The competitive effect of individual *i* with phenotype *z_i_* on individual *j* with phenotype *z_j_* follows the Gaussian function:

(4) $$\alpha \;\left(z_{i},z_{j}\right)=\;\exp\left(-\frac{{\left(z_{i}-z_{j}\right)}^{2}}{2\sigma_{\alpha }^{2}}\right),$$

where σ_α_ determines the width of the competition function on *z*. When σ_α_ is large individuals with different phenotypes compete strongly for resources, and when σ_α_ is small individuals with different phenotypes compete weakly. The total strength of competition experienced by individual *i* is:

(5) $$A\;\left(z_{i}\right)=\sum_{j}\alpha \left(\;z_{i},z_{j}\right).$$

The probability that a juvenile with phenotype *z_i_* survives frequency dependent selection follows a Beverton–Holt function:

(6) P surv zi=1+r−1KziAzi−1,

where *r* > 1 is the population growth rate at low density. This parameterization of competition follows many previous models (e.g., Dieckmann and Doebeli 1999; Doebeli and Dieckmann 2000; Bolnick 2004, 2006; Doebeli 2005; Gilman and Behm 2011; Thibert‐Plante and Hendry 2011).

Individuals that survive viability selection enter the mating phase. Each female evaluates a set of randomly selected males according to their ecological phenotypes, and chooses one male as a mate. A female's relative preference for male *i* with phenotype *z_i_* is described by the Gaussian function:

(7) $$\psi_{i}=\;\exp\left(-\frac{c^{2}}{2}{\left(z_{i}-p\left(p_{t},p_{a}\right)\right)}^{2}\right),$$

where *c* is her choosiness and *p* describes her preferred male phenotype (see Eq. (2)). The number of males that each female evaluates is drawn independently from a Poisson distribution with mean 20, but is capped at 10% of the male population. Limiting the number of males that each female evaluates is biologically realistic (Kokko and Brooks 2003), and it limits the strength of sexual selection in small populations. The probability that a female chooses male *i* from the set *M* she evaluates is:

(8) Pi,M=ψiψi∑j∈Mψj∑j∈Mψj.

Every female mates exactly once, regardless of her choosiness. Males can mate once, more than once, or not at all. Thus, choosiness by females exerts sexual selection on males, but females do not experience sexual selection or incur costs of choosiness.

Mated females produce offspring that form the pool of juveniles in the next generation. The number of offspring each female produces is drawn independently from a Poisson distribution with mean 2*r*. Offspring inherit one allele from each parent at each locus, with free recombination between loci. Each ecological or mate preference allele mutates with probability μ*_z_*, and each choosiness allele mutates with probability μ*_c_*. If a mutation occurs, a random quantity is added to the parental allele. This quantity is drawn from a distribution *N*(0, $\delta_{z}^{2}$) for ecological and mate preference alleles or *N*(0, $\delta_{c}^{2}$) for choosiness alleles. If a mutation causes the magnitude of an ecological allele to exceed ζ_max_, then the allele value is rounded to ‐ζ_max_ (if the allele is negative) or ζ_max_ (if the allele is positive). Biologically, this means that there is a maximum effect that any QTL can have on the ecological phenotype.

### ANALYSIS 1: INITIAL SPECIATION

We simulated evolving populations to study the potential for ecological speciation under unbiased and biased mate choice strategies. We initialized our model with randomly mating populations in which the mate choice strategy we wished to study could arise due to mutations at choosiness loci. Initial populations comprised 10^3^ individuals with each ecological or mate preference allele drawn independently from *N*(0, $\delta_{z}^{2}$) and each choosiness allele set to zero. We iterated 10^4^ generations under stabilizing ecological selection, with mutations allowed at ecological and mate preference loci, but not at choosiness loci. This burn‐in process removed the effect of initialization before choosiness began to evolve. Then, we altered the resource distribution so that ecological selection became disruptive. This change in the selective regime might represent the invasion of a new habitat (Losos 2009) or a change in the population's native habitat (Grant and Grant 2014). We iterated 10^5^ generations under the new selective regime with mutations allowed at all loci, and we asked whether choosiness evolved and assortative mating split the population into reproductively isolated groups. We called two groups reproductively isolated if females in each group were 10 times more likely to accept potential mates from their own group than from the other group (Thibert‐Plante and Gavrilets 2013), and we called this speciation if it persisted for 10^3^ generations. We recorded the time to speciation as the first of these 10^3^ consecutive generations. All parameter values are presented in the Supporting Information.

### ANALYSIS 2: REPEATED SPECIATION

During adaptive radiations, lineages speciate rapidly and repeatedly (Schluter 2000; Allender et al. 2003; Gavrilets and Losos 2009; Losos 2009). Thus, if a model captures the mechanisms of adaptive radiation, a population that has speciated once should be able to speciate again. Moreover, if evolved traits (e.g., mate choice strategies) make lineages prone to speciation, then respeciation should be faster than initial speciation. We used respeciation trials to ask whether the mate choice strategies we studied produce rapid repeated speciation events, and so might explain adaptive radiation.

Respeciation trials began with populations that had recently speciated. We obtained recently speciated populations by extracting daughter species from simulations that speciated in analysis 1. We randomly selected five males and five females from each daughter species to create one founder population per daughter species. We created 100 founder populations for each combination of conditions that produced speciation events in analysis 1. If a combination of conditions produced more than 100 daughter species, we used only 100 daughter species to create founder populations. If a combination produced fewer than 100 daughter species, we created multiple founder populations from each daughter species as necessary to reach 100 total founder populations. We used each founder population to initialize new simulations (i.e., respeciation trials). The values of all parameters in each respeciation trial were identical to those under which the founder population had evolved. Thus, a respeciation trial might simulate a population colonizing a new island without competition but with an environment similar to that in which the founders evolved (e.g., island colonization by Carribean anoles, Losos 2009). Founder populations were not optimally suited to their new environments because they had evolved to partition resources with sister species that were not present in the new environment. Thus, we expected the populations to evolve, and we expected selection to favor ecological divergence and respeciation. We iterated 10^5^ generations in each respeciation trial. We recorded whether each population respeciated, and if so we recorded the time to respeciation.

## Results

### ANALYSIS 1: INITIAL SPECIATION

When mate preferences are acquired without bias, phenotype matching, maternal imprinting, and less frequently paternal imprinting and genetic preferences can permit ecological speciation (Fig. 1C). Speciation is faster under modes that speciate more frequently (Table S2). Bias away from a learned phenotype increases the probability of speciation under each mate preference mode (compare Fig. 1D to C). Bias promotes speciation by modifying the effect of sexual selection on the ecological phenotype. Before populations begin to diverge, most females learn or have genetically determined preferences for common target phenotypes. If mate preferences are unbiased, females seek mates that exactly match these targets. This creates sexual selection against rare phenotypes, and opposes ecological divergence and speciation (Kirkpatrick and Nuismer 2004). If preferences are biased, then choosy females prefer mates with phenotypes slightly away from their target phenotypes. This weakens (or, if nongenetic variability in the ecological phenotype is sufficiently small, reverses) the stabilizing effect of sexual selection on the ecological phenotype, which facilitates divergence and promotes speciation (see also Fig. S1). Results are similar when the avoided phenotype is learned in ways other than by oblique imprinting (Table S2).

Biased learning changes which mate preference modes most strongly promote speciation. When mate preferences are unbiased, maternal imprinting promotes speciation more strongly than paternal imprinting (Fig. 1C). This is because all adult females become mothers, but males must be chosen as mates to become fathers. Thus, males but not females experience sexual selection. Because sexual selection is initially stabilizing, the set of fathers includes a narrower range of phenotypes than the set of mothers, and paternally imprinting females imprint on this narrower set. This creates stronger stabilizing selection on the ecological phenotype under paternal imprinting, and inhibits divergence and speciation. When mate preferences are biased, paternal imprinting becomes a stronger promoter of speciation than maternal imprinting (Fig. 1D). Bias removes the stabilizing effect of sexual selection before divergence. Once populations begin to diverge, paternal imprinting is better than maternal imprinting at maintaining reproductive isolation between the diverging groups. Under paternal imprinting, females prefer mates with the same extreme phenotypes that their mothers chose. If males with intermediate phenotypes arise (by mutation or hybridization), they fail to mate and therefore no females imprint on them. In contrast, females with intermediate phenotypes do reproduce. Thus, under maternal imprinting, some females imprint on and prefer intermediate phenotypes, which promotes hybridization and inhibits speciation.

### ANALYSIS 2: REPEATED SPECIATION

**Figure 2:**
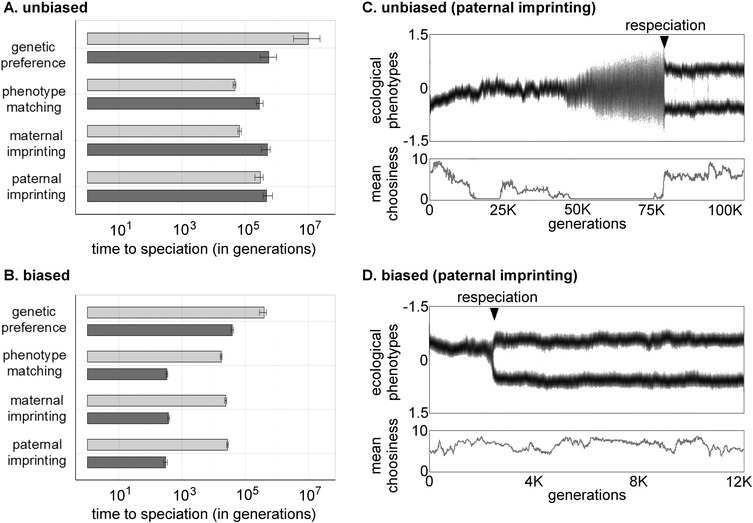
Biased mate preferences promote rapid repeated speciation. Left panels show median times to speciation (light bars) and respeciation (dark bars) under each mate preference mode when mate preferences are unbiased (A) or biased away from an obliquely imprinted phenotype (B). Under phenotype matching and parental imprinting, respeciation is slower than speciation when mate preferences are unbiased (A) but faster when preferences are biased (B). Under all mate preference modes, respeciation is up to two orders of magnitude faster when preferences are biased than when they are unbiased (compare dark bars in B to those in A). Note that *x*‐axes are on the log scale. Results are based on 1000 simulations per mate preference mode. Error bars show bootstrapped 90% confidence intervals. Results presented are for σ*_e_* = 0.06, but results are similar for other values of σ*_e_* (Supporting Information). Right panels show representative respeciation events under unbiased (C) and biased (D) paternal imprinting. Dark (light, white) areas represent ecological phenotypes at high (low, zero) density. Triangles indicate the point at which respeciation occurs. Lines in the lower panels show the mean strength of choosiness in the population over time.

In addition to promoting initial speciation, biased mate preference learning facilitates rapid repeated speciation (Fig. 2). When mate preferences are unbiased and acquired by phenotype matching or parental imprinting, respeciation is up to eight times slower than initial speciation (Fig. 2A). When the same modes include a bias, respeciation is up two orders of magnitude faster than initial speciation (Fig. 2B). The difference in the time to respeciation under biased and unbiased mate preferences is due to differences in the nature of sexual selection. Because strong mate preferences evolve during speciation processes, females in recently speciated founder populations are very choosy in mating. When mate preferences are learned without bias, this choosiness creates stabilizing sexual selection on male ecological phenotypes, and prevents the founders’ phenotype from diverging. For respeciation to occur, choosiness in the population must be lost due to genetic drift, and then the randomly mating population can diverge and speciate de novo (Fig. 2C). When preferences are learned with bias, bias away from common ecological phenotypes makes sexual selection disruptive, and respeciation occurs while choosiness remains strong (Fig. 2D). Thus, the choosiness that evolves during an initial speciation event primes the population for respeciation if learned mate preferences are biased, but inhibits respeciation if learned mate preferences are unbiased. Results are similar for other modes of biased learning (Table S3).

When mate preferences are genetic, respeciation is faster than initial speciation even if the preferences are unbiased (Fig 2A). However, if mate preferences are genetic and biased, respeciation is more than an order of magnitude faster than if they are genetic and unbiased (compare Fig. 2A to B). Moreover, bias increases the range of biologically plausible conditions under which genetic preferences can lead to speciation and respeciation (Fig. 1). Thus, even when female target phenotypes are genetically determined, bias away from a learned phenotype promotes rapid repeated speciation.

## Discussion

This study provides the first evidence that biased mate preference learning promotes rapid repeated ecological speciation and enables adaptive radiation. In contrast, when mate preferences do not include biases, repeated ecological speciation within a lineage is slow and adaptive radiation is inhibited. Evolutionary biologists have speculated that lineages can evolve traits or attributes that allow them to speciate more readily (Schluter 2000; Coyne and Orr 2004; Beltman and Metz 2005; Seehausen 2006; Losos 2009; Hugall and Stuart‐Fox 2012). Our results show that biased mate preference learning is one such trait. Thus, biased learning may play an important and hitherto underappreciated role in the generation of animal biodiversity.

In the past 20 years, mate preference learning has been shown to be widespread in vertebrates (e.g., zebra finches, Verzijden et al. 2007; cichlids, Verzijden et al. 2008; sticklebacks, Kozak et al. 2011; Darwin's finches, Grant and Grant 2014) and invertebrates (e.g., wolf spiders, Hebets 2003; fruit flies, Dukas 2004; crickets, Bailey and Zuk 2009; damselflies Svensson et al. 2010). Several of these lineages have undergone recent adaptive radiations (e.g., cichlids, Verzijden et al. 2008; finches, ten Cate et al. 2006; Verzijden et al. 2007; Grant and Grant 2014; sticklebacks, Schluter 2000). Our results suggest that the association between mate preference learning and adaptive radiation is not haphazard. Bias in mate preference learning has not been well‐studied in most lineages. More empirical work on diversified and less diversified lineages will help to test the role of biased mate preference learning in adaptive radiation.

Many adaptive radiations in nature involve the repeated filling of the same ecological niches. For example, three‐spined sticklebacks have diverged to fill benthic and limnetic niches at least four times (Schluter 2000), and Caribbean anoles have diverged to fill the same small set of niches on multiple islands (Losos 2009). Even when the parallel evolution of ecotypes is less apparent, much of the initial divergence between incipient species often begins along a single ecological trait axis. For example, Galapagos finches have repeatedly diverged in beak size (Grant and Grant 2014), and speciation among the African cichlids often begins with divergence in nuptial coloration (Allender et al. 2003). Our model incudes a single ecological trait axis, and captures this critical first step in adaptive radiation. Reproductively isolated populations might subsequently diverge in other ways. We have not attempted to capture that in our model.

Some previous models have produced adaptive radiations without biased mate preferences. Some of these models generate polytomies (Bolnick 2006) or rely on habitat choice instead of mate preference to maintain reproductive isolation (Gavrilets and Vose 2005). Others require that ecological divergence begin in allopatry (Aguilee et al. 2011). Our study is the first to explain the rapid sequential evolution of reproductive isolation by assortative mating without allopatry, as appears to have occurred in many adaptive radiations in nature (Schluter 2000; Allender et al. 2003; Losos 2009; Grant and Grant 2014).

In addition to facilitating adaptive radiation, bias changes the mate preference modes that most strongly promote ecological speciation. In particular, bias can make paternal imprinting a stronger driver of speciation than maternal imprinting. Yeh and Servedio (2015) showed that even unbiased paternal imprinting can strongly promote speciation if both the mate preference and the target phenotype are learned (as in some bird song). Both our model and Yeh and Servedio's (2015) assume polygynous mating systems. Paternal imprinting is plausible in polygynous systems if females raise offspring in the territories of the males they have chosen (e.g., great reed warblers, Hasselquist et al. 1996; dickcissels, Sousa and Westneat 2013) or if males raise their young from multiple broods (e.g., sticklebacks, Whoriskey and Fitzgerald 1994). Theory suggests that paternal imprinting is likely to evolve in systems where females can accurately identify their fathers (Tramm and Servedio 2008; Chaffee et al. 2013; Invernizzi and Gilman 2015).

Our model assumes that choosiness evolves without costs. That is, choosy females are not less likely to survive or mate than randomly mating females. In nature, choosiness may be costly (Kopp and Hermisson 2008; Otto et al. 2008). For example, choosier females may reject more potential mates, and so may miss some opportunities to reproduce. In addition, the sensory and neurological apparatus needed to exercise choosiness may be energetically expensive, and investing in this apparatus may mean individuals invest less in survival and reproduction. Biologically reasonable costs can inhibit the evolution of choosiness, but are not expected to prevent the evolution of choosiness or assortative mating in general (Beltman and Metz 2005; Doebeli 2005; Kopp and Hermisson 2008; Otto et al. 2008; Chaffee et al. 2013). We do not expect that reasonable costs of choosiness will alter the qualitative predictions of our model.

Our study demonstrates the evolutionary effects of different mate choice strategies that exist in nature, but we have not attempted to determine which of these strategies should evolve. Evolutionarily stable strategies for acquiring target phenotypes (but not for acquiring biases) have been studied elsewhere (Tramm and Servedio 2008; Chaffee et al. 2013; Invernizzi and Gilman 2015). In nature, biased mate preference learning might evolve due to selection (e.g., bias away from same‐sex parents might help individuals more accurately or efficiently identify the correct sex for courtship) or it might be a nonadaptive by‐product of the way sensory systems are formed (ten Cate and Rowe 2007). The origin of biased learning is an important question that must be resolved empirically. The results presented here assume that females bias their mate preferences away from obliquely imprinted phenotypes. This is motivated by the observation that females in nature often assess potential mates relative to other males they have encountered (Gibson and Langen 1996; Rebar et al. 2011). In the Supporting Information, we show that results are qualitatively similar if females shift their preference away from other learned phenotypes (e.g., parental phenotypes). In contrast, if the avoided phenotype is innate (e.g., genetically determined rather than learned), then biases do not promote speciation.

Researchers have argued that mate preference learning may play an important role in speciation (Verzijden et al. 2012). Our results show that biased mate preference learning can promote speciation under a broad range of biologically plausible conditions. Moreover, biased learning greatly increases the probability of repeated speciation, and thus of adaptive radiation. Thus, biased mate preference learning may play an important, but previously unrecognized role in generating and maintaining animal biodiversity.

## Supporting information


**Figure S1**. Mating success of males as a function of ecological phenotype (*z*) and female choosiness in the population (κ).
**Figure S2**. A representative respeciation process when mate preference is genetic and unbiased.
**Figure S3**. Speciations per 100 simulations when mate preferences are genetic and (A) unbiased or (B) biased away from an obliquely imprinted phenotype.
**Table S1**. Variables used in this study.
**Table S2**. Probability of initial speciation and median time to speciation under different positive and negative mate preference modes.
**Table S3**. Probability of respeciation and median time to respeciation under different positive and negative mate preference modes.
**Table S4**. Speciations per 100 simulations (top) and median time to speciation (bottom) under different combinations mutation rate (μ*_z_*), ecological allele effect size (ζ*_z_*), and expected magnitude of mutations to choosiness alleles (δ*_c_*) both with and without a bias away from an obliquely imprinted phenotype (*s*_max_ = 0.12, *m* = 2).
**Table S5**. Respeciations (top) per 100 trials and median time to respeciation (bottom) under different combinations mutation rate (μ*_z_*), ecological allele effect size (ζ*_z_*), and expected magnitude of mutations to choosiness alleles (δ*_c_*) both with and without a bias away from an obliquely imprinted phenotype (*s*_max_ = 0.12, *m* = 2).
**Table S6**. Probabilities of speciation under different combinations of positive and negative mate preference modes, using three different definitions of speciation.
