# Supplementary material for: Learning to speciate: The biased learning of mate preferences promotes adaptive radiation
Source: Evolution. 2015 Oct 26;69(11):3004–12. doi: 10.1111/evo.12797 (PMC5057300; doi:10.1111/evo.12797)
Supplement: Supplementary file 1 — Figure S1. Mating success of males as a function of ecological phenotype (z) and female choosiness in the population (κ). Figure S2. A representative respeciation process when mate preference is genetic and unbiased. Figure S3. Speciations per 100 simulations when mate preferences are genetic and (A) unbiased or (B) biased away from an obliquely imprinted phenotype. Table S1. Variables used in this study. Table S2. Probability of initial speciation and median time to speciation under different positive and negative mate preference modes. Table S3. Probability of respeciation and median time to respeciation under different positive and negative mate preference modes. Table S4. Speciations per 100 simulations (top) and median time to speciation (bottom) under different combinations mutation rate (μz), ecological allele effect size (ζz), and expected magnitude of mutations to choosiness alleles (δc) both with and without a bias away from an obliquely imprinted phenotype (s max = 0.12, m = 2). Table S5. Respeciations (top) per 100 trials and median time to respeciation (bottom) under different combinations mutation rate (μz), ecological allele effect size (ζz), and expected magnitude of mutations to choosiness alleles (δc) both with and without a bias away from an obliquely imprinted phenotype (s max = 0.12, m = 2). Table S6. Probabilities of speciation under different combinations of positive and negative mate preference modes, using three different definitions of speciation. [file EVO-69-3004-s001.docx]

**ONLINE SUPPORTING INFORMATION**

**Contents**

1. Parameter values
2. Assessing reproductive isolation and measuring time to speciation
3. Figures illustrating the effects of sexual selection on the ecological phenotype in populations with unbiased and biased mate preferences
4. Mechanisms not discussed in the body of the paper
5. Results when females learn to avoid phenotypes other than obliquely imprinted phenotypes
6. Results obtained using different parameter values
7. Additional references

**I. Parameter values**

The variables we used and the values we assigned to them in our simulations are presented in Table S1. Parameter values affect the absolute probabilities and rates of speciation, but do not affect our qualitative results (section VI). We chose the width of the competition function as the scaling parameter for our model, and we set this to *σ_α_* = 0.5. Other parameter values (*e.g*., the width of the resource distribution *σ_z_*, the magnitude of mutations at ecological trait loci *δ_z_*, and the nongenetic variability in the ecological phenotype *σ_e_*) can be interpreted relative to *σ_α_*.

*Parameter values that describe the population*

We set the reproductive rate in our model to *r* = 4, which is within the range found in nature (Korpimaki 1992). We set the mutation rate at ecological loci to *μ_z_* = 3.125 x 10^-4^, so that one out of every 100 individuals experiences a mutation somewhere in its genome that affects its ecological phenotype. We set the mutation rate at choosiness loci to *μ_c_* = 1.25 x 10^-4^, so that one out of every 1000 individuals experiences a mutation that affects its choosiness. The lower genome-wide mutation rate at choosiness loci reflects our assumption that QTLs affecting choosiness are smaller or rarer than QTLs affecting the ecological phenotype. We set the mutation magnitudes to *δ_z_* = 0.025 and *δ_c_* = 1. This means that most mutations affecting the ecological phenotype have small effects, but some mutations that affect choosiness are large. Large mutations to choosiness alleles favor speciation (Rettelbach et al. 2011), so choosing large *δ_c_* allows us to study cases in which speciation is likely. We controlled the maximum effect size of ecological QTLs by setting *ζ_max_* = 0.077. This is important because, if ecological QTL sizes are allowed to evolve freely under disruptive selection, all genetic variability becomes concentrates at a single locus (Kopp and Hermisson 2006), but in nature ecological phenotypes are usually controlled by multiple QTLs (Rockman 2012). When *ζ_max_* = 0.077, there will be genetic variability in at least 4 loci in an optimally diverged population under disruptive selection in our model. In simulations with biased mate preferences, we set the maximum bias to *b_max_* = 0.12 and the rate at which bias declines with an increasing difference between a female’s target and avoided phenotypes to *m_b_* = 2. Thus, bias in our model is small rather than open-ended (see *Describing the magnitude of bias* below).

*Parameter values that describe the environment*

The environment in our model is described by the resource distribution function *K*(*z*). To generate stabilizing selection, we drew the optimal ecological phenotype, *z^*^*, from a uniform distribution *U*(-1,1) and we set the width of the resource distribution to *σ_z_* = 0.5, the kurtosis to *β* = 2, and the maximum to *K*(*z^*^*) = 3440. Under these parameter values, selection on the ecological phenotype is stabilizing at *z^*^* and genetic variability at ecological trait loci is maintained by mutation. To generate disruptive selection, we set *z^*^* = 0, *σ_z_* = 1, *β* = 4, and *K*(*z^*^*) = 2000. These parameter values create disruptive selection that cannot be resolved in a randomly mating population (Doebeli et al. 2007), and so favor ecological speciation. The different values of *K*(*z^*^*) under stabilizing and disruptive selection ensure that the total amount of resources in the system is the same before and after the change in the selective regime.

*Describing the magnitude of bias*

Under the parameter values we studied, the standard deviation of the ecological phenotype in a randomly mating adult population in the steady state under disruptive selection is approximately 0.42. The maximum bias in mate preference (*b_max_*) is approximately 28% of the standard deviation of the ecological phenotype, and bias declines to zero when the target and avoided phenotypes differ by 1.2 standard deviations. The expected bias for target and avoided phenotypes drawn randomly from the population is 0.038, or 8.9% of the standard deviation of the ecological phenotype.

**II. Assessing reproductive isolation and measuring time to speciation**

To determine whether reproductive isolation evolved in a given simulation, we assayed matings between individuals with different ecological phenotypes every 100 generations. If the population could be divided at some point on the *z* axis into two groups such that females in each group were 10 times more likely to accept mates from their own group than from the other group, then we said the groups were reproductively isolated in that generation. If groups remained reproductively isolated for at least 10^3^ generations (*i.e*., 10 or more consecutive assays), then we said that speciation had occurred (Thibert-Plante and Gavrilets 2013). We recorded the time to speciation as the first of the 10 or more consecutive assays in which we observed reproductive isolation. If speciation occurred in the first 10^3^ generations of a simulation, we assayed the population every 10 generations and recorded the time to speciation as the first of 100 or more consecutive assays with reproductive isolation. This gave us greater resolution for very fast speciation events. Other definitions of speciation yield qualitatively similar results (section VI).

If speciation occurred under a given combination of conditions, then we wanted a measure of how quickly it occurred. We used the median time to speciation, because it is less sensitive than the mean speciation time to rare slow speciation events. If more than half of the simulations under a given combination of conditions speciated, we simply observed the median speciation time. If fewer than half of the simulations speciated, we predicted the median speciation time from the data.

*Predicting median time to speciation*

Consider a set of *k* simulations, each of *g* generations. Assume that *n* of *k* simulations speciate. The speciation events occur in generations {*t_1_*, *t_2_*, …, *t_n_*} where *t_1_* ≤ *t_2_* ≤ … ≤ *t_n_*. Let *t_x+1/2_* = (*t_x_* + *t_x+1_*)/2 for all *x* ∈ ℤ. If *n* > *k*/2, then the median time to speciation, *M*, is simply *t_(k+1)/2_*, and we can observe this in the data. If *n* ≤ *k*/2, then we cannot observe *M* in the data and we must predict *M*.

In our simulations, speciation rates declined over time. To predict the median speciation time, we used the last 10 observed speciation events (or all speciation events if there were fewer than 10) to estimate the speciation rate at the end of the simulation. We assumed that this rate would not change after the end of the simulation, and we used it to calculate the expected time to the median speciation event.

Let *ρ* be the instantaneous rate of speciation. Assume that *ρ* is constant over the last 10 speciation events and does not change after generation *g*. If *n* ≤ 10, then *ρ* is constant in generations 1 to *g*, and the expected proportion of simulations that speciates by generation *g* is 1 - *e^-ρg^*. The observed proportion of simulations that speciated by generation *g* is *n*/*k*. Thus, we estimate *ρ* using

|  | (S2.1) |
| --- | --- |

If *n* > 10, then the last 10 speciation events occur in the last *g* – *t_n-10_* generations. The proportion of unspeciated simulations that speciate in this time is 10/(*k* – *n* + 10). We estimate *ρ* using

|  | (S2.2) |
| --- | --- |

If *n* ≤ *k*/2 in generation *g*, then the median speciation event will be the ((*k* + 1)/2 – *n*)^th^ speciation after generation *g*. If simulations speciate at rate *ρ*, then the expected time of the median speciation event will be

| . | (S2.3) |
| --- | --- |

If speciation rates were still declining at the end of our simulations, then this approach will have overestimated the final speciation rate and thus underestimated the median speciation time. Moreover, it will have underestimated the median speciation time by more for populations that speciate more slowly. Because our goal is to identify differences between speciation rates and this approach underestimates those differences, it is conservative with respect to the question we studied.

**III. Figures illustrating the effects of sexual selection on the ecological phenotype in populations with unbiased and biased mate preferences**

The different effects of unbiased and biased mate preferences on ecological speciation are due to how sexual selection acts on the ecological phenotype in evolving populations. In particular, the mating success of males with a given ecological phenotype depends on whether female preferences are unbiased or biased. When preferences are unbiased, strong choosiness by females favors common male phenotypes and so exerts stabilizing selection on the ecological phenotype. When preferences are biased, this sexual selection becomes disruptive. Here, we illustrate the effects of unbiased and biased mate preferences on male mating success. In order to compare male mating success under unbiased and biased mate preference strategies, we must control the strength of choosiness and the distribution of ecological phenotypes in the populations we are studying. We cannot do this in stochastic simulations. Therefore, we developed a mathematical approximation of our model and we studied it numerically.

Consider a population in which the ecological phenotype of adults is normally distributed with mean zero and variance *σ*^2^. The relative density of adults with ecological phenotype *z* will be

| $g\left( z \right)=\frac{1}{\sqrt{2\pi\sigma^{2}}}\text{exp}\left( -\frac{z^{2}}{2\sigma^{2}} \right).$ | (S3.1) |
| --- | --- |

Assume that the population is sexually monomorphic, so *g*(*z*) describes the distribution of ecological phenotypes in both males and females. Furthermore, assume that each female acquires a target phenotype by maternal imprinting and an avoided phenotype by oblique imprinting. Thus, the distribution of target and avoided phenotypes, *t*(*z*) and *a*(*z*) respectively, will be the same as the distribution of ecological phenotypes (*i.e*., *t*(*z*) = *a*(*z*) = *g*(*z*)), and each female’s target and avoided phenotypes will be independent. The density of females with target phenotype *x* and avoided phenotype *y* will be

| $f\left( x,y \right)=\frac{1}{\sqrt{2\pi\sigma^{2}}}\text{exp}\left( -\frac{x^{2}}{2\sigma^{2}} \right)\frac{1}{\sqrt{2\pi\sigma^{2}}}\text{exp}\left( -\frac{y^{2}}{2\sigma^{2}} \right).$ | (S3.2) |
| --- | --- |

Following Eqs. 1 and 2 in the main text, the mate preference of a female with target and avoided phenotypes *x* and *y*, respectively, will be

| $p\left( x,y \right)=x+\text{max}\left[ b_{max}\left( 1-m_{b}\left\vert x-y \right\vert\right),0 \right]\text{sgn}\left( x-y \right).$ | (S3.3) |
| --- | --- |

Following Eq. 7, the relative preference of a female with target and avoided phenotypes *x* and *y*, respectively, for a male with phenotype *z* is

| $\psi\left( x,y,z \right)=\text{exp}\left( -\frac{\kappa^{2}}{2}\left( z-p\left( x,y \right) \right)^{2} \right),$ | (S3.4) |
| --- | --- |

where $\kappa$ indicates the strength of choosiness.

For mathematical convenience, we assume that every female assesses the entire male population before selecting a mate. This means that the effect of $\kappa$ on choosiness will be quantitatively stronger than, but qualitatively similar to, the effect of *c* on choosiness in our stochastic simulations. The probability that a female with target and avoided phenotypes *x* and *y*, respectively, selects a mate with ecological phenotype *z* is the relative probability that she accepts a male with phenotype *z*, multiplied by the density of that phenotype in the population, and scaled for the fact that each female selects exactly one mate. We can write this

| $P\left( z\vert x,y \right)=\frac{\psi\left( x,y,z \right)g\left( z \right)}{\int_{-\infty}^{\infty} \psi\left( x,y,\zeta\right)g\left( \zeta\right) d\zeta}.$ | (S3.5) |
| --- | --- |

The expected mating success of a male with ecological phenotype *z* is the probability that a male with that phenotype is selected by a female with target and avoided phenotypes *x* and *y*, summed over all combinations of *x* and *y* in the female population, and then divided by the density of males with that phenotype. We can write this

| $E\left( z \right)=\frac{\int_{-\infty}^{\infty} \int_{-\infty}^{\infty} P\left( z\vert x,y \right)f\left( x,y \right) dx dy}{g(z)}.$ | (S3.6) |
| --- | --- |

We can evaluate Eq. S1.6 for different combinations of parameter values. Fig. S1 shows the expected mating success of males as a function of the ecological phenotype, *z*, the choosiness of females, *κ*, and the standard deviation of ecological phenotypes in the population, *σ*. As choosiness evolves, the value of *σ*  in the population changes, because some males fail to mate. In Figs. S1A and B, we set *σ* = 0.42. This is the approximate distribution of phenotypes in our stochastic simulations when ecological selection is disruptive and mating is random (*i.e*., *c* = 0). In Figs. S1C and D, we set *σ* = 0.08. This is the approximate distribution of phenotypes in our stochastic simulations when ecological selection is disruptive and choosiness has begun to evolve (*i.e*., *c* ≥ 3) but when ecological phenotypes have not yet begun to diverge. In each case, we estimated *σ* in populations with parameter values reported in Table S1 and with *σ_e_* = 0.06.

Fig. S1 illustrates how biased mate preferences promote speciation. If mate preferences are unbiased, speciation is possible only if ecological phenotypes diverge while choosiness is still weak (*i.e*., at low values of *κ* in Fig. S1A). If choosiness becomes strong before the ecological phenotypes have diverged (*i.e*., high values of *κ* in Fig. S1C), then stabilizing sexual selection overcomes disruptive ecological selection and prevents speciation. Moreover, once choosiness becomes strong, choosiness is stable. This is because females with weaker choosiness are more likely to accept mates with rare ecological phenotypes and so to have sons with rare ecological phenotypes, and those sons have low mating success. In contrast to Fig. S1C, if mate preferences are biased (*e.g*., in Fig. S1D), sexual selection is disruptive when choosiness is strong (*i.e*., high values of *κ* in Fig. S1D). This promotes divergence and speciation in populations where strong choosiness has already evolved.

Fig. S1 also explains why biased mate preferences promote respeciation but unbiased mate preferences inhibit respeciation. During an initial speciation process, daughter species evolve strong choosiness. If mate preferences are unbiased (high values of *κ* in Fig. S1C), this choosiness inhibits divergence and respeciation in the daughter species. If mate preferences are biased (high values of *κ* in Fig. S1D), this choosiness promotes divergence and respeciation.

**IV. Mechanisms not discussed in the body of the paper**

*1. Phenotype matching and parental imprinting promote ecological speciation more strongly than genetic preferences, and oblique imprinting does not promote ecological speciation (Fig. 1C).*

Speciation in our model occurs when populations evolve to mate assortatively. This happens when individuals choose mates with phenotypes (and therefore genotypes) similar to their own. Speciation is easiest under phenotype matching because phenotype matching is the most direct mechanism of assortative mating. Under maternal and paternal imprinting, females prefer mates that resemble their parents rather than themselves. Females often have phenotypes similar to their parents, so females that imprint on their parents tend to prefer mates with phenotypes similar to their own. However, because this mechanism of assortment is less precise than phenotype matching, parental imprinting promotes speciation less strongly. Genetic preferences cause assortative mating only when the mate preference genotype is positively correlated with the ecological phenotype in the population. Such correlations can evolve, but they can also dissipate rapidly due to genetic recombination during mating, and if this happens divergence that has already emerged will disappear. This makes genetic preference a slower and less reliable mechanism of speciation than phenotype matching (as previously argued by Doebeli 2005) or parental imprinting. Oblique imprinting causes females to imprint on males that may or may not be similar to themselves, and so cannot cause assortative mating or speciation.

The difference between genetic preferences and the other mate preference modes can be put in the context of Felsenstein’s (1981) one-allele and two-allele speciation mechanisms (e.g., Albert 2005). Phenotype matching and parental imprinting are one-allele mechanisms of speciation. That is, speciation occurs when the same alleles (*i.e.*, choosiness alleles) become fixed in each incipient species. Genetic preference is a two-allele mechanism of speciation, because it requires that different alleles (*i.e*., alleles coding for different target phenotypes) become fixed in each incipient species. In Felsenstein’s models, one-allele mechanisms are stronger promoters of speciation than two-allele mechanisms. Our results show this remains true when one-allele mechanisms involve mate preference learning.

Interestingly, paternal imprinting is a weaker promoter of speciation than maternal imprinting. This is due to stabilizing sexual selection acting on the ecological phenotype in males. In our model, mothers include all females that have survived viability selection in each generation, but fathers include only those males that have first survived viability selection and then been chosen as mates by females. Females that prefer mates with common phenotypes are likely to find them, but females that prefer mates with rare phenotypes may fail to find them and accept less preferred common males instead. As a result, sexual selection favors common male phenotypes and reduces the relative density of rare phenotypes among fathers. This means that phenotypes in the paternal imprinting set (*i.e*., the set of fathers; Tramm and Servedio 2008) are less variable than those in the maternal imprinting set (*i.e*., the set of mothers). Speciation requires that different females prefer different ecological phenotypes. As a result, ecological speciation is easier when the imprinting set is more variable (as in maternal imprinting) than when it is less variable (as in paternal imprinting).

Using a model in which choosiness was already present in the population and did not need to evolve, Verzijden and colleagues (2005) found that phenotype matching and parental imprinting promoted speciation more strongly than genetic preferences. In a model where choosiness was an evolvable trait, Beltman and Metz (2005) found that learned habitat preferences promoted speciation more strongly than genetic habitat preferences. Our study is the first to show that learned mate preferences promote speciation more strongly than genetic preferences when choosiness in mating must evolve *de novo*. The evolution of choosiness in mating is likely to be an important step in ecological speciation in nature (Schluter 2000; Coyne and Orr 2004; Nosil 2012). Thus, our model provides the strongest evidence to date for the long-standing conjecture that mate preference learning can promote speciation (Immelmann 1975; Laland 1994; Irwin and Price 1999; Verzijden et al. 2012).

*Nongenetic variability in the ecological phenotype reduces the probability of speciation. Even small amounts of nongenetic variability prevent ecological speciation when mate preferences are genetic (Fig. 1C).*

Nongenetic variability in the ecological phenotype inhibits speciation because it reduces the correspondence between phenotype and genotype. Under phenotype matching and parental imprinting, females are less likely to choose mates with genotypes similar to their own if the phenotypes they learn to prefer and the phenotypes they use to assess mates do not accurately reflect genotypes. This effect is compounded when mate preferences are genetic. If the phenotypes that females prefer in mates do not accurately predict the genotypes of the mates they choose, then correlations between ecological and mate preference genotypes in the population are slow to develop and quick to disappear. Thus, genetic preferences rarely result in assortative mating or speciation when there is nongenetic variability in the ecological phenotype. Nongenetic variability in ecological traits in nature is common and can be large (e.g., Angilletta et al. 2004; Teplitsky and Millien 2014). If this variability prevents speciation when mate preferences are genetic, then learned mate preferences may be critical to speciation in many systems.

*When mate preferences are biased, paternal imprinting promotes speciation more strongly than the other modes (Fig. 1D).*

Biased away from learned phenotypes changes which mate preference modes most strongly promote ecological speciation. In particular, when nongenetic variability in the ecological phenotype is large, biased learning makes paternal imprinting a stronger driver of speciation than either phenotype matching or maternal imprinting. This is true because biased mate preferences make sexual selection disruptive, and break the stabilizing selection that inhibits speciation when paternal imprinting is unbiased (see Fig. S1). Once the population begins to diverge, paternal imprinting is better than either of the other modes at maintaining reproductive isolation. To understand this, consider a rare hybridization event under maternal or paternal imprinting. Under maternal imprinting, F1 hybrid females seek mates with extreme phenotypes like their mothers, but the backcross females in the next generation seek mates with intermediate phenotypes like their F1 hybrid mothers. Thus, once begun, hybridization persists. In contrast, under paternal imprinting, F1 hybrid females seek mates with extreme phenotypes like their fathers, and backcross females learn to prefer the same extreme phenotypes their mothers preferred. Because hybrid males rarely find mates (due to sexual selection), few females imprint on intermediate male phenotypes, and hybrid females are quickly reabsorbed into the parental populations.

The difference between the effects of biased paternal and biased maternal imprinting on speciation can be placed in the context of nongenetic inheritance (Danchin et al. 2004). Under paternal imprinting, females learn to prefer the same mate phenotypes that their mothers selected. Thus, they inherit their mate preferences from their mothers, independent of the genes that they inherit. Under maternal imprinting, a daughter’s mate preference can be different from her mother’s, and the preferred mate phenotype is not inherited. Thus, in the case of biased parental imprinting, the nongenetic inheritance of mate preferences promotes ecological speciation.

*Respeciation is faster than speciation when mate preferences are genetic and unbiased (Fig 2A).*

When mate preferences in a founder population are due to unbiased phenotype matching or paternal imprinting, those preferences are drawn from the set of ecological phenotypes already present in the population. Therefore, most females prefer mates with common phenotypes. This creates stabilizing sexual selection on the ecological phenotype, and prevents that phenotype from diverging. Because the ecological phenotype cannot diverge, neither can mate preferences. Thus, the founder phenotype is strongly stabilized as long as the population remains choosy, and choosiness in the founder population inhibits respeciation (Fig. 2C).

If mate preferences are unbiased and genetic, sexual selection still stabilises the ecological phenotype. However, in this case, new mate preference phenotypes can arise by mutations at mate preference loci. Mate preference phenotypes are less strongly stabilised than ecological phenotypes, and mate preferences can diverge. As mate preferences diverge, ecological phenotypes can also diverge, even while the founder population remains choosy (Fig. S2). Thus, choosiness in the founder population prior to invasion promotes respeciation, and respeciation is faster than initial speciation.

**V. Results when females learn to avoid phenotypes other than obliquely imprinted phenotypes**

*Rationale and approach*

In the main text, we assumed that biased mate preferences are shifted away from the phenotype of randomly encountered males (*i.e*., avoided phenotypes are obliquely imprinted). In nature, females may learn their avoided phenotypes in other ways (ten Cate et al. 2006; ten Cate and Rowe 2007; Rutledge et al. 2010). For example, a female zebra finch will seek a mate with a beak color similar to her father’s and avoid mates with beak colors similar to her mother’s. Thus, her mate preference is shifted away from her father’s phenotype in the direction opposite her mother’s phenotype (ten Cate et al. 2006).

Here we study how rates of speciation and respeciation depend on how females learn avoided phenotypes. We consider cases in which the avoided phenotype is genetically determined (*genetic*) and in which it is learned. A female can learn to avoid her own phenotype (*phenotype matching*), her mother’s phenotype (*maternal imprinting*), her father’s phenotype (*paternal imprinting*), the phenotype of an adult male she selects at random (*oblique imprinting*), or the population mean phenotype (*population mean imprinting*). We refer to the mode by which females acquire target phenotypes as the *positive mode*, and the mode by which they acquire avoided phenotypes as the *negative mode*. If the negative mode is genetic, we assume that the avoided phenotype is determined by 16 additive diploid loci, each of which houses one of an infinite number of real-valued alleles. In nature, individuals often assess mates relative to other potential mates in the population (Gibson and Langen 1996; Rutledge et al. 2010; Rebar et al. 2011). The oblique negative mode represents an extreme case in which females assess potential mates relative to a single alternative mate. The population mean negative mode represents the other extreme, in which females assess mates relative to all other males in the population.

To study the effect of different positive and negative modes on speciation and respeciation, we conducted 125 speciation trials and 125 respeciation trials for each combination of positive and negative mode, and 125 speciation and 125 respeciation trials for each positive mode when it is unbiased. We conducted trials as in the body of the paper, and we set parameter values as in Table S1. We focused on the case where *σ_e_* = 0.06 (*i.e*., nongenetic variability in the ecological phenotype is small). We chose *σ_e_* = 0.06 because we wanted to include respeciation under genetic preferences in our results. Speciation did not occur under unbiased genetic preferences when *σ_e_* > 0.06, so we could not study respeciation in these cases.

*Results*

Bias away from each of the learned avoided phenotypes that we studied promotes ecological speciation (Table S2). Initial speciation is most frequent and fastest when females avoid the population mean phenotype. Avoiding the population mean phenotype causes females to choose mates with ecological phenotypes shifted away from the population mean in the same direction as their own. This makes sexual selection disruptive and results immediately in assortative mating. The oblique negative mode also strongly promotes initial speciation. Phenotypes learned by oblique imprinting are surrogates, albeit imprecise ones, for the population mean, and promote speciation in the same way. The maternal, paternal and phenotype matching negative modes cause females to avoid phenotypes similar to their own. This does not produce immediate assortative mating, but it increases the variability in mate preferences in the population. When different females prefer different ecological phenotypes, the stabilizing effect of sexual selection is relaxed. Males with rare phenotypes that are favored by ecological selection can sometimes mate, and thus ecological phenotypes in the population can diverge. As ecological phenotypes diverge, even females with some bias in their mate preferences tend to select mates with phenotypes relatively similar to their own. This allows speciation to occur.

Bias away from learned phenotypes also promotes rapid repeated speciation (Table S3). When mate preferences are acquired by unbiased phenotype matching or unbiased parental imprinting, respeciation is several times slower than initial speciation (Table S3, column 1). When mate preferences are biased away from learned phenotypes, respeciation is up to two orders of magnitude faster than initial speciation (Table S3, columns 3-7). Biased mate preferences promote respeciation by reversing stabilizing sexual selection in founder populations (Fig. S1). When this happens, choosiness already present in the founder population promotes rather than inhibits population divergence, and respeciation occurs while choosiness remains strong. This means that a population that has evolved choosiness during an earlier speciation event has evolved the ability to speciate rapidly in the future. As a result, bias in mate preferences enables adaptive radiation.

Interestingly, when combined with learned positive modes, the genetic negative mode inhibits rather than promotes respeciation (Table S3, column 2). Founder populations have evolved genetic biases to protect them from interbreeding with their sister species. The preference of every female in the founder population is shifted in the same direction (away from the sister species), and thus sexual selection in the founder population is directional rather than disruptive or stabilising. This inhibits adaptation to the new environment. Moreover, conflicting ecological and sexual selection stabilize the ecological phenotype in the founder population and inhibit population divergence and respeciation.

Finally, when both the target and avoided phenotypes are genetically determined, respeciation is faster than initial speciation (Table S3). In this case, sexual selection stabilises the ecological phenotype but does not strongly stabilize the genetic mate preference of the founder population. Therefore, mate preferences can diverge. As mate preferences diverge, the stabilising effect of sexual selection on the ecological phenotype is broken, and the ecological phenotype can also diverge. Nonetheless, respeciation when both the positive and negative modes are genetic is up to several orders of magnitude slower than when the positive and negative modes are learned.

**VI. Results obtained under different parameter values**

Results presented in this paper were obtained by studying stochastic individual-based simulations. Their generality is limited by the need to assign values to model parameters. In the main text, we present simulations using the parameter values in Table S1. Here we show that results are qualitatively similar for other biologically reasonable parameter sets. In particular, we study 1) other mutation rates, effect sizes of ecological alleles, and effect sizes of choosiness alleles; 2) genetic preferences that are controlled by fewer than 16 loci; and 3) other definitions of speciation.

*1) Speciation and respeciation rates under other mutation rates and allele effect sizes*

The process of ecological speciation is sensitive to mutation rates (Gavrilets 2005; Polechova and Barton 2005), the effect size of ecological alleles (Gavrilets 2004; Thibert-Plante and Gavrilets 2013), and the effect size of choosiness alleles (Rettelbach et al. 2011). In particular, low mutation rates and small allele effect sizes inhibit ecological speciation. Here we show that biased learning promotes ecological speciation and permits rapid repeated speciation even when mutation rates are low and/or allele effect sizes are small. Thus, biased learning increases the range of biologically plausible conditions under which ecological speciation can occur, and promote adaptive radiation under a wide range of plausible conditions.

We conducted 125 speciation trials under each of 144 combinations of mate preference mode, mutation rate at ecological loci (*μ_z_*), ecological allele effect size (*ζ_z_*), and magnitude of mutations to choosiness alleles (*δ_c_*), both with and without a bias away from an obliquely imprinted phenotype (18,000 total simulations). Under the mutation rates we studied, the probability that an individual has a mutation somewhere its genome that affects its ecological phenotype is either 0.01 (*μ_z_* = 3.125 x 10^-4^) or 0.001 (*μ_z_* = 3.125 x 10^-5^). Under the ecological allele effect sizes we studied, two optimally diverged species have different alleles at at least 1 (*ζ_z_* = 0.31), 4 (*ζ_z_* = 0.077), or 8 (*ζ_z_* = 0.038) loci. Controlling the magnitude of mutations to choosiness alleles allowed us to control the effect size of choosiness alleles in our model. Smaller mutations produce alleles with smaller effects. In each speciation trial we set *σ_e_* = 0.06 and all other parameters as in Table S1. If speciation occurred two or more times under a given set of conditions, we created founder populations from the daughter species and conducted 125 respeciation trials under the same set of conditions (11,375 total simulations).

Tables S4 and S5 show the results of speciation and respeciation trials, respectively. As in previous studies, lower mutation rates (compare cell columns 1 and 2 with cell columns 3 and 4 in each table), smaller effect sizes of ecological alleles (compare entries left to right within cells), and smaller mutations to choosiness alleles (compare entries bottom to top within cells) inhibit speciation. The tables support the following results from the body of the paper:

*Speciation*

1. Speciation is slower and less frequent when mate preferences are genetic than when they are learned (compare cell rows in Table S4).
2. Speciation is faster and more frequent when mate preferences are biased than when they are unbiased (compare cell column 1 with cell column 2, and cell column 3 with cell column 4 in Table S4).

*Respeciation*

1. When mate preferences are learned without bias, respeciation is slower than initial speciation (compare cell columns 1 and 3 in table S4 with cell columns 1 and 3 in Table S5).
2. When mate preferences are learned with bias, respeciation is faster than initial speciation (compare cell columns 2 and 4 in Table S4 with cell columns 2 and 4 in Table S5).
3. When mate preferences are genetic, respeciation is faster than initial speciation (compare cell row 1 in Table S4 with cell row 1 in Table S5). But, if mate preferences are genetic and biased away from a learned phenotype, respeciation is up to two orders of magnitude faster than if mate preferences are genetic and unbiased (compare the cell in row 1 column 3 to the cells in column 4 of Table S5).

*Speciation rates when nongenetic preferences are governed by fewer than 16 loci*

In the main text, we showed that ecological speciation is more sensitive to nongenetic variability in the ecological phenotype when mate preferences are genetic than when they are acquired by phenotype matching or parental imprinting. There, we assumed that genetic preferences are governed by 16 independently assorting loci, but in nature genetic preferences may be governed by fewer loci (Haesler and Seehausen 2005). Here we show that speciation is more sensitive to nongenetic variability when mate preferences are controlled by fewer loci. Because genetic preferences are less likely to lead to speciation when the number of loci controlling those preferences is small, results presented in the body of this paper may overestimate the ability of genetic preferences to cause speciation, and underestimate the importance of biased learning in adaptive radiation.

We conducted 125 speciation trials when genetic preferences were governed by 1, 2, 4, 8, or 16 loci with additive effects, and when the nongenetic variability was in the set *σ_e_* = {0, 0.03, … 0.30}. We did this for both unbiased preferences and for preferences biased away from an obliquely imprinted phenotype (13,750 total simulations) with parameters set as in Table S1.

Fig. S3 shows the number of speciations per 100 simulations when preferences are (A) unbiased or (B) biased. Speciation is more difficult when genetic preferences are governed by fewer loci. For all numbers of preference loci, bias increases the probability of speciation (compare Fig. S3A to S3B). Moreover, for all numbers of preference loci, speciation is more sensitive to nongenetic variability when mate preferences are genetic than when they are acquired by phenotype matching or parental imprinting (compare Fig. S3 to Fig. 1).

*Speciation rates obtained using different speciation thresholds*

In the body of this paper, we called two subpopulations “species” if females from each subpopulation were 10 times (10x) more likely to accept mates from their own subpopulation than to accept mates from the other subpopulation. We chose this threshold to be consistent with other studies of ecological speciation (Thibert-Plante and Gavrilets 2013), but we could have chosen a higher or lower threshold instead. Table S6 shows how choosing different speciation thresholds affects speciation probabilities in our study. In particular, the table shows the probabilities of speciation under different combinations of positive and negative mate preference modes when speciation requires females to be 5x, 10x, or 100x more likely to accept mates from their own subpopulation. For most mode combinations, the probability of speciation does not depend on the speciation threshold. This is because speciation processes that reach the 5x threshold rapidly progress until reproductive isolation is complete or nearly complete. However, when the positive mode is parental imprinting and the negative mode is parental imprinting or phenotype matching (cells outlined in red), speciation processes that reach the 10x threshold sometimes fail to reach the 100x threshold.

To understand why this is true, consider a population in which the ecological trait is beak size. Assume that the positive and negative mate preference modes are paternal and maternal imprinting, respectively. Assume that the population has reached the 10x speciation threshold, so that there are small-beaked and large-beaked subpopulations. Within the large-beaked subpopulation, most females choose mates with beaks larger than their own. The daughters of these females imprint on their fathers’ phenotypes and shift their preferences away from their mothers’ phenotypes. Therefore, they also tend to choose mates with beaks larger than their own. This promotes reproductive isolation. However, some very large-beaked females necessarily accept mates with beaks smaller than their own. The daughters of these females look for mates with beaks smaller than their fathers’. This causes a small amount of hybridization and prevents reproductive isolation from reaching the 100x threshold.

The results presented here show that many combinations of positive and negative mate preference modes promote the evolution of reproductive isolation, but reproductive isolation can reach different levels of completeness under different combinations. In particular, parental imprinting promotes stronger reproductive isolation when it is biased away from the population mean or away from an obliquely imprinted phenotype (which acts a as a surrogate for the population mean) than when it is biased away from the other parent’s phenotype or away from the individual’s own phenotype. Nonetheless, mate preference modes that produce incomplete reproductive isolation may facilitate speciation if reduced gene flow allows the build-up of genetic incompatibilities between subpopulations. In such cases reinforcement may complete the speciation process (Coyne and Orr 2004). A similar result might arise if sexual dimorphism were to evolve (Bolnick and Doebeli 2003). For example, if males evolve phenotypes more extreme than females, paternal imprinting with bias away from the maternal phenotype could result in strong reproductive isolation.

**Table S1. Variables used in this study.**

| **Name** | **Definition** | **Assigned value** |
| --- | --- | --- |
| *Variables describing individual phenotypes* | | |
| *z* | ecological phenotype | genetically controlled with random nongenetic error *σ_e_* |
| *p^•^* | ecological phenotype preferred in mates (females only) | genetically controlled or learned |
| *p_t_* | target ecological phenotype in mates | “ |
| *p_a_* | avoided ecological phenotype in mates (females in simulations with biased mate preference acquisition only) | “ |
| *c* | choosiness in mating (females only) | genetically controlled |
| *Parameters controlling genetic architecture and population dynamics* | | |
| *μ_z_* | per locus mutation rate at ecological and mate preference QTLs | 3.125 x 10^-4^ |
| *μ_c_* | per locus mutation rate at choosiness QTLs | 1.25 x 10^-4^ |
| *δ_z_* | expected magnitude of effect of mutations in ecological and mate preference QTLs | 0.025 |
| *δ_c_* | expected magnitude of effect of mutations in choosiness QTLs | 1 |
| *ζ_max_* | limits the effect size of ecological QTLs | 0.077 |
| *σ_e_* | expected nongenetic effect on the ecological phenotype | various |
| *r* | maximum rate of population growth | 4 |
| *Parameters controlling the resource distribution and competition* | | |
| *z** | optimal ecological phenotype for a monomorphic population (Eq. 3) | *U*(-1,1) (stabilizing selection) or 0 (disruptive selection) |
| *K*(*z**) | carrying capacity of juveniles in an optimally adapted monomorphic population (Eq. 3) | 3400 (stabilizing selection) or 2000 (disruptive selection) |
| *σ_z_* | controls the width of the resource distribution (Eq. 3) | 0.5 (stabilizing selection) or 1.0 (disruptive selection) |
| *β* | kurtosis of the resource distribution (Eq. 3) | 2 (stabilizing selection) or 4 (disruptive selection) |
| *σ_α_* | controls strength of competition between phenotypically dissimilar individuals (Eq. 4) | 0.5 |
| *Parameters controlling bias in mate preference* | | |
| *b_max_* | maximum bias (Eq. 1) | 0.12 |
| *m_b_* | controls how bias declines with \|*p_t_ - p_a_*\| (Eq. 1) | 2 |

**Table S2. Probability of initial speciation and median time to speciation under different positive and negative mate preference modes**. In each cell, the probability of speciation within 10^5^ generations is listed above and the median time to speciation (in 1000s of generations) is listed below. Green shading indicates that the combination of positive and negative modes results in significantly faster speciation than an unbiased preference with the same positive mode (significance tested by Monte Carlo randomization). Speciation is more frequent and faster when mate preferences are biased away from a learned phenotype. Results are based on 125 simulations per mode combination with parameters set as in Table S1 and *σ_e_* = 0.06.

|  |  |  | | **negative mode** | | | | | |
| --- | --- | --- | --- | --- | --- | --- | --- | --- | --- |
|  |  | unbiased | genetic | | phenotype matching | maternal imprinting | paternal imprinting | oblique imprinting | population mean imprinting |
| **positive mode** | genetic preference | 0.01  9,870 | 0.08  717 | | 0.05  1,430 | 0.02  2,890 | -  - | 0.16  398 | 0.50  103 |
|  | phenotype matching | 0.66  46.5 | 0.66  54.6 | |  | 0.88  18.1 | 0.92  20.2 | 0.91  17.5 | 0.94  14.4 |
|  | maternal imprinting | 0.57  67.1 | 0.58  65.6 | | 0.88  24.8 |  | 0.88  23.2 | 0.86  24.0 | 0.88  21.8 |
|  | paternal imprinting | 0.29  297 | 0.30  189 | | 0.71  42.9 | 0.82  35.2 |  | 0.84  26.9 | 0.87  20.4 |

**Table S3. Probability of respeciation and median time to respeciation under different positive and negative mate preference modes**. In each cell, the probability of respeciation within 10^5^ generations is listed above and the median time to respeciation (in 1000s of generations) is listed below. Green (red) shading indicates that respeciation is significantly faster (slower) than speciation for that combination of positive and negative modes (Monte Carlo randomization; compare table S3 to table S2). Respeciation is more frequent and faster when mate preferences are biased away from a learned phenotype. Results are based on 125 simulations per mode combination with parameters set as in Table S1 and *σ_e_* = 0.06.

|  |  |  | | **negative mode** | | | | | |
| --- | --- | --- | --- | --- | --- | --- | --- | --- | --- |
|  |  | unbiased | genetic | | phenotype matching | maternal imprinting | paternal imprinting | oblique imprinting | population mean |
| **positive mode** | genetic preference | 0.18  553 | 0.58  34.6 | | 0.54  69.6 | 0.33  220 | - | 0.78  39.1 | 0.98  0.25 |
|  | phenotype matching | 0.27  233 | 0.22  230 | |  | 0.99  0.14 | 0.98  0.24 | 0.99  0.44 | 1.00  0.24 |
|  | maternal imprinting | 0.18  345 | 0.16  331 | | 1.00  0.05 |  | 1.00  0.03 | 0.99  0.33 | 1.00  0.16 |
|  | paternal imprinting | 0.10  390 | 0.06  1,060 | | 1.00  0.15 | 0.98  0.05 |  | 1.00  0.28 | 1.00  0.16 |

**Table S4.** Speciations per 100 simulations (top) and median time to speciation (bottom) under different combinations mutation rate (*μ_z_*), ecological allele effect size (*ζ_z_*), and expected magnitude of mutations to choosiness alleles (*δ_c_*) both with and without a bias away from an obliquely imprinted phenotype (*s_max_* = 0.12, *m* = 2). The presence of biased learning is indicated by the “yes” and “no” columns. Median times to speciation are in 1000s of generations. Empty cells indicate that no speciation events occurred under these parameter combinations. Each entry is based on 125 simulations.

|  | *μ_z_* | 3.125 x 10^-5^ | | | | | | 3.125 x 10^-4^ | | | | | |  |
| --- | --- | --- | --- | --- | --- | --- | --- | --- | --- | --- | --- | --- | --- | --- |
|  | bias | No | | | Yes | | | No | | | Yes | | |  |
|  | *ζ_z_* | 0.31 | 0.077 | 0.038 | 0.31 | 0.077 | 0.038 | 0.31 | 0.077 | 0.038 | 0.31 | 0.077 | 0.038 |  |
| genetic preference | | *δ_c_*=2 |  |  |  |  |  | 13  283 | 6  1,060 | 7  938 | 6  1,220 | 16  426 | 53  94.7 | 65  42.1 |
|  |  | *δ_c_*=1 |  |  |  |  |  | 2  4,350 | 2  4,350 | 1  8,730 | 2  4,350 | 2  2,890 | 16  304 | 26  228 |
|  |  | *δ_c_*=0.5 |  |  |  |  |  |  |  |  |  | 1  8,730 | 2  4,350 | 7  938 |
| phenotype matching | | *δ_c_*=2 | 2  2,890 |  |  | 68  31.6 | 67  32.0 | 74  30.7 | 83  10.5 | 71  42.3 |  | 98  4.1 | 95  6.8 | 83  15.3 |
|  |  | *δ_c_*=1 | 2  4,350 |  |  | 49  104 | 46  119 | 42  146 | 85  8.8 | 60  64.5 |  | 96  6.7 | 89  15.3 | 57  62.2 |
|  |  | *δ_c_*=0.5 |  |  |  | 17  370 | 12  393 | 16  277 | 91  12.0 | 68  54.1 |  | 94  10.3 | 81  35.1 | 33  152 |
| maternal imprinting | | *δ_c_*=2 |  |  |  | 74  46.2 | 66  33.3 | 62  38.4 | 82  10.7 | 58  56.2 |  | 98  4.5 | 92  9.8 | 84  17.1 |
|  |  | *δ_c_*=1 | 1  8,730 |  |  | 48  107 | 50  96.9 | 34  190 | 80  15.7 | 48  114 |  | 94  9.1 | 90  29.8 | 50  96.6 |
|  |  | *δ_c_*=0.5 |  |  |  | 13  443 | 11  444 | 10  496 | 87  15.8 | 53  85.4 |  | 94  13.5 | 77  46.3 | 22  262 |
| paternal imprinting | | *δ_c_*=2 |  |  |  | 73  30.4 | 67  31.0 | 72  31.3 | 81  18.2 | 42  134 |  | 94  5.5 | 94  8.8 | 73  19.5 |
|  |  | *δ_c_*=1 |  |  |  | 53  92.6 | 46  114 | 42  142 | 80  20.9 | 32  175 |  | 95  10.5 | 87  23.2 | 54  77.1 |
|  |  | *δ_c_*=0.5 | 1  8,730 |  |  | 18  338 | 16  350 | 14  367 | 62  41.7 | 6  1,220 |  | 94  16.5 | 52  91.4 | 28  192 |

**Table S5.** Respeciations (top) per 100 trials and median time to respeciation (bottom) under different combinations mutation rate (*μ_z_*), ecological allele effect size (*ζ_z_*), and expected magnitude of mutations to choosiness alleles (*δ_c_*) both with and without a bias away from an obliquely imprinted phenotype (*s_max_* = 0.12, *m* = 2). The presence of biased learning is indicated by the “yes” and “no” columns. Median times to respeciation are in 1000s of generations. Empty cells indicate that fewer than two speciation events occurred under these parameter combinations. Each entry is based on 125 simulations.

|  | *μ_z_* | 3.125 x 10^-5^ | | | | | | 3.125 x 10^-4^ | | | | | |
| --- | --- | --- | --- | --- | --- | --- | --- | --- | --- | --- | --- | --- | --- |
|  | bias | No | | | Yes | | | No | | | Yes | | |
|  | *ζ_z_* | 0.31 | 0.077 | 0.038 | 0.31 | 0.077 | 0.038 | 0.31 | 0.077 | 0.038 | 0.31 | 0.077 | 0.038 |
| genetic preference | *δ_c_*=2 |  |  |  |  |  | 50  102 | 34  163 | 29  202 | 18  458 | 26  365 | 85  17.0 | 98  2.5 |
|  | *δ_c_*=1 |  |  |  |  |  | 10  442 | 26  295 | 26  289 | 13  629 | 28  332 | 82  18.0 | 94  2.8 |
|  | *δ_c_*=0.5 |  |  |  |  |  |  |  |  |  |  | 60  67.6 | 70  22.2 |
| phenotype matching | *δ_c_*=2 | 1  8,730 |  |  | 96  0.59 | 98  0.60 | 97  0.63 | 46  122 | 31  199 |  | 100  0.13 | 100  0.12 | 100  0.13 |
|  | *δ_c_*=1 | 2  4,350 |  |  | 93  0.89 | 97  0.70 | 96  0.87 | 43  116 | 25  229 |  | 100  0.37 | 99  0.25 | 98  0.35 |
|  | *δ_c_*=0.5 |  |  |  | 90  2.3 | 90  3.6 | 95  2.0 | 28  199 | 15  348 |  | 100  0.99 | 94  3.9 | 89  0.74 |
| maternal imprinting | *δ_c_*=2 |  |  |  | 100  0.53 | 99  0.40 | 100  0.50 | 34  173 | 21  304 |  | 100  0.07 | 100  0.07 | 100  0.09 |
|  | *δ_c_*=1 |  |  |  | 96  0.89 | 98  0.96 | 95  0.80 | 34  163 | 14  375 |  | 100  0.23 | 99  0.24 | 94  0.42 |
|  | *δ_c_*=0.5 |  |  |  | 91  2.4 | 89  4.4 | 94  4.2 | 19  300 | 10  422 |  | 98  0.90 | 97  0.90 | 95  0.60 |
| paternal imprinting | *δ_c_*=2 |  |  |  | 99  0.14 | 99  0.20 | 98  0.12 | 32  210 | 14  381 |  | 100  0.03 | 100  0.04 | 97  0.03 |
|  | *δ_c_*=1 |  |  |  | 94  0.60 | 97  0.73 | 94  0.42 | 38  157 | 6  1,220 |  | 100  0.24 | 99  0.20 | 97  0.25 |
|  | *δ_c_*=0.5 |  |  |  | 94  1.8 | 92  1.3 | 92  0.89 | 20  202 | 2  4,350 |  | 99  2.0 | 95  0.91 | 89  0.70 |

**Table S6. Probabilities of speciation under different combinations of positive and negative mate preference modes, using three different definitions of speciation**. In each cell, the top, middle, and bottom numbers are the probabilities of speciation within 10^5^ generations when speciation requires that females are 5x, 10x, or 100x more likely to choose mates from their own species. Red boxes highlight mode combinations under which reproductive isolation sometimes evolves to only intermediate (10x) strengths. Results in each cell are based on the same simulations reported in Table S2.

|  |  |  | | **negative mode** | | | | | |
| --- | --- | --- | --- | --- | --- | --- | --- | --- | --- |
|  |  | unbiased | genetic | | phenotype matching | maternal imprinting | paternal imprinting | oblique imprinting | population mean |
| **positive mode** | genetic preference | 0.01  0.01  0.01 | 0.08  0.08  0.08 | | 0.05  0.05  0.05 | 0.01  0.01  0.01 | -  -  - | 0.16  0.16  0.15 | 0.50  0.50  0.50 |
|  | phenotype matching | 0.66  0.66  0.65 | 0.66  0.66  0.64 | |  | 0.88  0.88  0.86 | 0.92  0.92  0.90 | 0.91  0.91  0.91 | 0.94  0.94  0.94 |
|  | maternal imprinting | 0.57  0.57  0.56 | 0.58  0.58  0.57 | | 0.88  0.88  0.01 |  | 0.88  0.88  0.40 | 0.86  0.86  0.86 | 0.88  0.88  0.88 |
|  | paternal imprinting | 0.29  0.29  0.28 | 0.30  0.30  0.29 | | 0.71  0.71  0.63 | 0.82  0.82  0.66 |  | 0.84  0.84  0.84 | 0.93  0.93  0.93 |

**Figure S1. Mating success of males as a function of ecological phenotype (*z*) and female choosiness in the population (*κ*).** Female mate preferences are acquired by unbiased (**A**, **C**) or biased (**B**, **D**) maternal imprinting. Panels **A**, **B** assume that male ecological phenotypes in the population are unimodally distributed with high variability (*σ* = 0.42). This occurs when choosiness in the population is weak. When choosiness is strong, sexual selection eliminates variability in the ecological phenotype (**C**, **D**, *σ* = 0.08). When choosiness is strong, unbiased mate preferences generate stabilizing sexual selection and inhibit speciation, but biased mate preferences generate disruptive sexual selection and promote speciation.

**Figure S2. A representative respeciation process when mate preference is genetic and unbiased.** In **A**, dark (light, white) areas represent phenotypes at high (low, zero) density. Gray indicates ecological phenotypes, and red (plotted under gray) indicates genetically determined mate preference phenotypes. The distribution of mate preferences is wider than the distribution of ecological phenotypes because mate preference is not as strongly stabilized by sexual selection. The black triangle indicates the point at which respeciation occurs. **B** shows the mean strength of choosiness in the population, and **C** shows the correlation between the ecological and mate preference phenotypes.

**Figure S3. Speciations per 100 simulations when mate preferences are genetic and (A) unbiased or (B) biased away from an obliquely imprinted phenotype.**

Speciations

Nongenetic variability (σ_e_)

B

Speciations

A

**VII. Additional references**

Albert, A. Y. K. 2005. Mate choice, sexual imprinting, and speciation: A test of a one-allele isolating mechanism in sympatric sticklebacks. Evolution 59:927-931.

Angilletta, M. J., T. D. Steury, and M. W. Sears. 2004. Temperature, growth rate, and body size in ectotherms: Fitting pieces of a life-history puzzle. Integr. Comp. Biol. 44:498-509.

Beltman, J. B. and J. A. J. Metz. 2005. Speciation: more likely through a genetic or through a learned habitat preference? Proc. R. Soc. Lond. 272:1455-1463.

Bolnick, D. I. and M. Doebeli. 2003. Sexual dimorphism and adaptive speciation: Two sides of the same ecological coin. Evolution 57:2433-2449.

Danchin, E., L. A. Giraldeau, T. J. Valone, and R. H. Wagner. 2004. Public information: From nosy neighbors to cultural evolution. Science 305:487-491.

Felsenstein, J. 1981. Skepticism Towards Santa Rosalia, or Why Are There So Few Kinds of Animals. Evolution 35:124-138.

Gavrilets, S. 2004. Fitness Landscapes and the Origin of Species. Princeton University Press, Princeton, NJ.

Gavrilets, S. 2005. "Adaptive speciation" - It is not that easy: A reply to Doebeli et al. Evolution 59:696-699.

Haesler, M. P. and O. Seehausen. 2005. Inheritance of female mating preference in a sympatric sibling species pair of Lake Victoria cichlids: implications for speciation. Proc. R. Soc. Lond. 272:237-245.

Immelmann, K. 1975. Ecological Significance of Imprinting and Early Learning. Annu. Rev. Ecol. Syst. 6:15-37.

Korpimaki, E. 1992. Fluctuating Food Abundance Determines the Lifetime Reproductive Success of Male Tengmalms Owls. J. Anim. Ecol. 61:103-111.

Laland, K. N. 1994. On the Evolutionary Consequences of Sexual Imprinting. Evolution 48:477-489.

Polechova, J. and N. H. Barton. 2005. Speciation through competition: A critical review. Evolution 59:1194-1210.

Rettelbach, A., J. Hermisson, U. Dieckmann, and M. Kopp. 2011. Effects of genetic architecture on the evolution of assortative mating under frequency-dependent disruptive selection. Theor. Popul. Biol. 79:82-96.

Rockman, M. V. 2012. The Qtn Program and the Alleles That Matter for Evolution: All That's Gold Does Not Glitter. Evolution 66:1-17.

Rutledge, J. M., A. Miller, and G. W. Uetz. 2010. Exposure to multiple sensory cues as a juvenile affects adult female mate preferences in wolf spiders. Anim. Behav. 80:419-426.

Teplitsky, C. and V. Millien. 2014. Climate warming and Bergmann's rule through time: is there any evidence? Evol Appl 7:156-168.
